# Threads of Whale Tendon as a Substitute for Catgut

**Published:** 1878-10

**Authors:** 


Threads of Whale Tendon as a Substitute for Catgut.—Baelf (*Centralb. fur Chirg.*) following Dr. Ischiguro, of Japan, the inventor, recommends these threads. From experiments, he states that they are more rapidly and more completely absorbed than catgut ligatures. He finds that this absorption is assisted by steeping them in oil of the whale's liver. In operations he finds them more flexible than catgut, and from experience, considers that the only objection that could be made to them for ligaturing arteries, viz., that the absorption would be too rapid, is unfounded, and besides, could be got over by torsion of the arteries.—*Archives of Clinical Surgery.*

